# Development of Sustained Release Chitosan/β-Glycerol Phosphate-Based Injectable Thermo-Gels Containing Naproxen Sodium: *In-Vitro* and *In-Vivo* Evaluation

**DOI:** 10.1007/s11095-026-04197-y

**Published:** 2026-09-22

**Authors:** Hassan Ahmad, Sadia Jafar Rana, Tahir Ali Chohan, Haashim Iqbal, Rawdha Benziane, Manoochehr Rasekh, Saman Zafar, Muhammad Sohail Arshad, Zeeshan Ahmad

**Affiliations:** 1 Faculty of Pharmacy, University of Central Punjab, Lahore, Pakistan; 2 Department of Pharmaceutics, Bahauddin Zakariya University, Multan, Pakistan; 3 The Next Institute of Science and Technology, Multan, Pakistan; 4 Institute of Pharmaceutical Sciences, University of Veterinary and Animal Sciences, Lahore, Pakistan; 5 Leicester School of Pharmacy, De Montfort University, Leicester, UK; 6 College of Engineering, Design and Physical Sciences, Brunel University of London, London, UK; 7 Division of Pharmacy and Optometry, School of Health Sciences, University of Manchester, Manchester, UK

**Keywords:** arthritis, inflammation, *in-situ* gel, sustained release, thermogelation

## Abstract

**Purpose:**

To develop *in-situ* chitosan/β-glycerol phosphate (β-GP) thermogels loaded with naproxen sodium for sustained drug delivery, to circumvent issues related with prolonged oral administration of naproxen (e.g. ulceration, abdominal discomfort, diarrhoea, bloating, etc.). To overcome these complications, *in-situ* gel formulations are developed as a promising strategy for long-term management of chronic inflammatory disorders such as arthritis. Furthermore, this system is evaluated with an earlier naproxen formulation prepared using sodium alginate.

**Methods:**

Thermogels containing varying chitosan concentrations were prepared at pH 7.4 and 37°C, followed by drug loading. Key physicochemical properties, including injectability, gelation time, and sol–gel fraction, were evaluated. *In-vitro* release studies were conducted using a dialysis membrane with a USP dissolution basket apparatus-I. Anti-inflammatory efficacy was assessed in Sprague–Dawley rats with carrageenan-induced hind ankle inflammation.

**Results:**

Results indicated that formulations with higher polymer content exhibited shorter gelation times and increased gel fractions. *In-vitro* release from F6CGP demonstrated sustained naproxen sodium delivery, following the Korsmeyer-Peppas model with Fickian diffusion kinetics (*n* ≤ 0.45). *In-vivo*, the swelling of the inflamed hind ankle joint decreased progressively over 72 h (≈ -2.6 ± 0.73 mm), confirming effective anti-inflammatory action; relatively better as compared to the previous sodium alginate system.

**Conclusions:**

*In-situ* chitosan/β-glycerol gel-forming injectable formulation F6CGP provides sustained naproxen sodium release and shows potential as a therapeutic strategy for chronic inflammatory conditions such as arthritis. The results also indicate that this polymeric system can play a key role in modulating critical formulation parameters i.e. gelation time, gel fraction, injectability, drug release etc.

## Introduction

Hydrogels are three-dimensional (3D) cross-linked polymeric networks formed from one or more monomers through relatively simple reactions. Composed of hydrophilic polymers, they exhibit remarkable water retention and swelling capacity, yet remain insoluble in water despite containing large amounts of it within their structure [1–4]. Based on the nature of their crosslinks, hydrogels are broadly categorised into synthetic hydrogels, where networks are stabilised by irreversible covalent bonds, and supramolecular hydrogels, where assembly relies on reversible non-covalent interactions such as hydrogen bonding and electrostatic forces. Certain hydrogels are stimuli-responsive, undergoing structural or functional changes in response to environmental factors. This makes them attractive choice as intelligent, *in situ*-forming carriers for the delivery of drugs, genes, proteins, and enzymes [5–8]. Owing to these unique properties, hydrogels have been widely explored for diverse applications including biosensing and tissue regeneration [9], bone repair [10, 11], biomedical engineering [12–16], and targeted drug delivery [17–20].

Due to their minimally invasive administration,* in situ*-forming injectable hydrogels hold significant promise for drug delivery. These systems are typically supramolecular or self-assembled hydrogels that are in sol or free-flowing state under physiological pH and room temperature, but undergo gelation upon contact with living tissues. The therapeutic agent can be encapsulated within the cross-linked networks, enabling enhanced retention and controlled release [21–24]. *In-situ* gelation can be triggered by several physiologic mechanisms including ions, pH and temperature [19, 25]. Some polymers (e.g., gelatin) can form gel via ionic bonds formation with carboxylic acid of amino acids (e.g., with calcium). Furthermore, incorporation of ions (e.g., calcium) into alginate can increase its viscosity via ionic crosslinking [26]. Polymers with pH sensitive properties (e.g., polyacrylate) transform from liquid to gel following a change in pH. Bonds between the polymers change due to a variation in pH leading to gel formation. Some polymers demonstrate *in-situ* gel formulations in response to pH changes. These comprise of acidic groups which undergo protonation at low pH of stomach and form a gel. Some polymers (e.g., poly(N-isopropyl acrylamide acid)) can undergo sol–gel transition following variations in temperature. Such polymers usually form a gel at a certain temperature known as the ‘Critical Point of Solution’. When such polymer solution is injected into the body, it undergoes a thermal change due to body temperature and forms gel [27]. By forming a localised depot, injectable hydrogels facilitate site-specific drug delivery while reducing systemic side effects and bypassing first-pass metabolism commonly associated with oral dosage forms [28]. Owing to their safety, cost-effectiveness, and ability to provide sustained and controlled release, injectable hydrogels are advantageous compared to conventional administration routes (e.g., oral, buccal, and sublingual). Consequently, they decrease dosing frequency and improve patient compliance.

Chitosan, a cationic polysaccharide of basic nature, is chemically composed of (1,4)−2-amino-2-deoxy-D-glucans. It is commercially produced by deacetylation of chitin, which is obtained from seafood waste. As the second most abundant natural polysaccharide after cellulose, chitosan is highly valued for biomedical applications. Its biodegradability into harmless amino sugars in humans makes it particularly suitable for wound healing, tissue regeneration and drug delivery [25, 29, 30]. Chitosan hydrogels can form via thermogelation at physiological pH, whereby the polymer solution transitions into a soft gel at body temperature (37°C) while remaining in sol form at ambient temperature [31, 32]. Chitosan-based *in-situ* gels are generally classified into two types: (i) covalent crosslinking systems, such as chitosan-genipin gels [33], and (ii) phase separation systems, such as chitosan/β-glycerophosphate (β-GP) gels. The latter relies on pH and temperature changes, along with solvent exchange, to generate electrostatic interactions, hydrogen bonding, and hydrophobic linkages [34].

Glycerol phosphate is an FDA-approved organic compound naturally present in the human body. As a phosphate source, it supports metabolic processes and can be administered intravenously. Beyond its metabolic role, β-GP has demonstrated significant efficacy in bone development when used as a supplement [34, 35]. Importantly, β-GP can neutralize acidic chitosan solution and induce its transformation into a thermosensitive gel under physiological pH and temperature.

Naproxen sodium (NAP) is an over-the-counter (OTC) nonsteroidal anti-inflammatory drug (NSAID) widely used for the treatment of arthritic and other inflammatory conditions. It is a non-selective cyclooxygenase (COX) inhibitor of the propionic acid derivative class. However, prolonged oral administration of NSAIDs such as NAP is associated with gastrointestinal side effects, including ulceration, abdominal discomfort, diarrhoea, bloating, and heartburn [36–38]. To mitigate these complications, *in-situ* gel formulations of NAP have been proposed as a promising strategy for long-term management of chronic inflammatory disorders such as arthritis.

The present study aimed to develop NAP-loaded *in situ*-forming injectable chitosan hydrogels, using β-GP as a gelling agent. The thermoresponsive hydrogels were evaluated for key physicochemical properties, including clarity, injectability, gelation time, sol–gel fraction, drying and hydration behavior, *in-vitro* drug release, and *in-vivo* anti-inflammatory activity. The hydrogels were also characterized using DSC, powder X-ray diffraction (pXRD) and ATR-FTIR spectroscopy. In addition, this study compares two *in-situ* gel systems viz, sodium alginate/calcium gluconate (ionic crosslinking) [26] and chitosan/β-GP (thermal gelation) under simulated physiologic and pathologic conditions. Since these systems rely on distinct gelation mechanisms, their physicochemical characteristics, gelation behavior, drug release profiles, and an* in vivo* anti-inflammatory study were compared to manifest usefulness in the treatment of arthritis.

## Materials and Methods

### Materials

Chitosan (CHIT; deacetylated chitin, medium molecular weight (MMW): 190,000–390,000 g/mol; degree of deacetylation: 75–85%; viscosity: 200–800 cps at 25 °C) and β- glycerol phosphate disodium salt pentahydrate (β-GP, ≥ 98.0%) were purchased from Sigma Aldrich (USA). Sodium hydroxide (NaOH) pellets were obtained from Uni-Chem Reagents, while potassium phosphate monobasic (KH_2_PO_4_; extra pure) was sourced from Duksan Reagents. Potassium hydrogen phthalate (C_8_H_5_KO_4_) was purchased from Merck (Germany). Naproxen Sodium (NAP) was provided as a gift sample by Divis Pharma Pvt. Ltd., and dialysis tubing with a molecular weight cut-off (MWCO) of 14,000 Da was obtained from Medicell Membranes Ltd. (UK). Fresh deionized water was prepared in-house. All other chemicals and solvents used in this study were of analytical grade.

### Methods

#### Preparation of *In-Situ* Injectable Chitosan/β-glycerol Phosphate (CGP) Gels

**Fig. 1:**
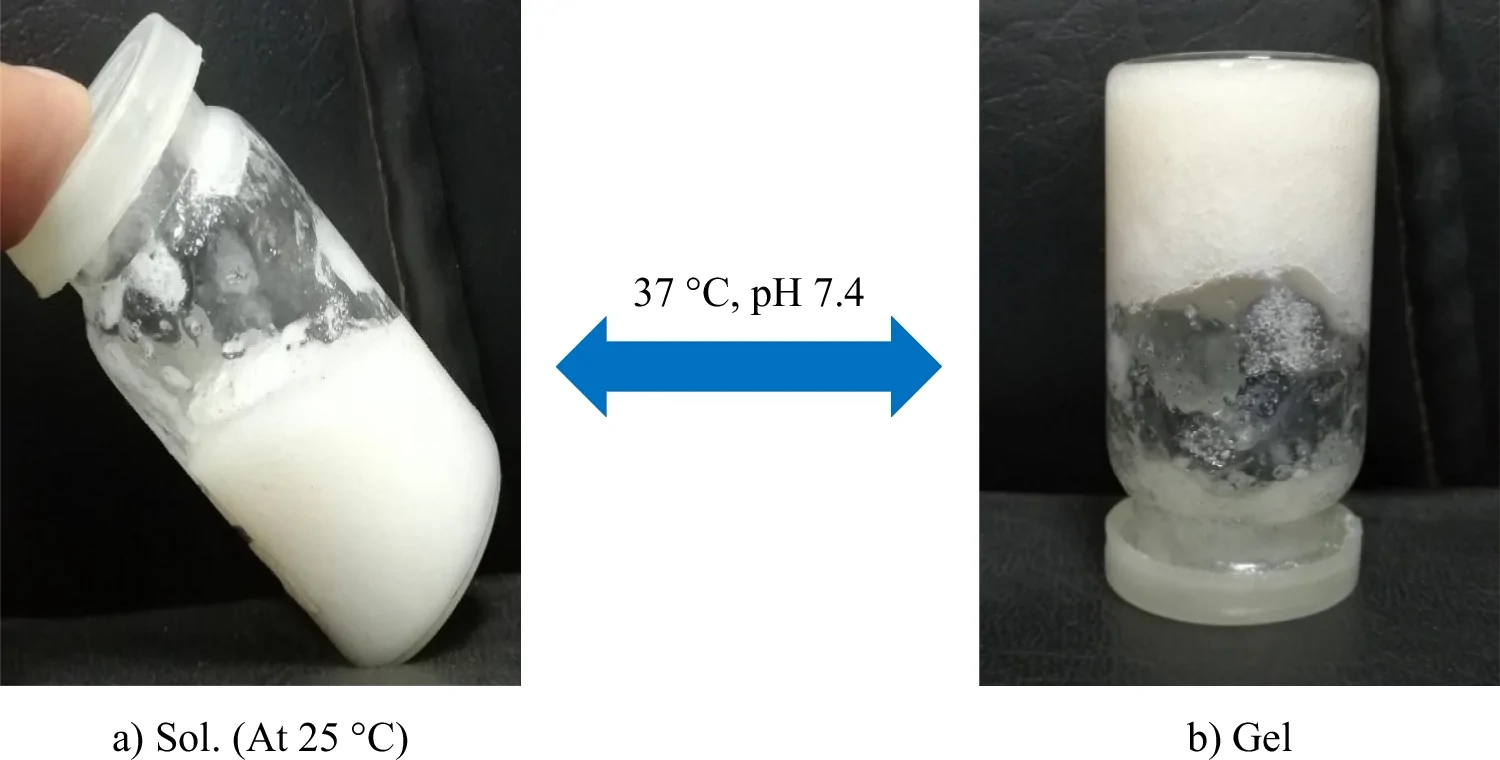
Sol–gel transition of chitosan/βGP (CGP) with naproxen sodium: (**a**) chitosan/βGP + naproxen sodium sol. (**b**) *in-situ* gel formed at 37 °C and pH 7.4.

Aqueous solutions of CHIT, β-GP, and NAP were prepared under a Class-I safety cabinet. Briefly, a weighed amount of chitosan was dissolved in 6 mL of 0.75% (v/v) acetic acid under vigorous stirring for 1 h. Separately, 100 mg of naproxen sodium was dissolved in 2 mL of deionized water, and 600 mg of β-GP was dissolved in another 2 mL of deionized water. The naproxen sodium solution was then added dropwise to the chitosan solution under continuous stirring at 250 rpm and stirred for 30 min. Subsequently, the β-GP solution was added dropwise to the mixture under constant stirring for an additional 1 h. A blank chitosan/β-GP (CGP) gel was prepared using the same procedure, except that 2 mL of deionized water was used instead of the naproxen sodium solution to serve as a control [39]. Polymer concentrations were varied as shown in Table 1, while the concentrations of β-GP and naproxen sodium were kept constant. All solutions were filtered through 0.45 μm nylon syringe filter. The pH of the formulations was measured and adjusted to 7.4. Finally, the solutions were placed in a water bath maintained at 37 ±  2 °C for 30 min to observe *in-vitro* gelation (Fig. 1) in flat-bottomed glass vials.

**Table 1:** Formulation composition for chitosan/β-GP (CGP) gels

Formulation codes	Ingredients		
	Chitosan (MMW) *(%w/v)*	β-glycerol-phosphate *(%w/v)*	Naproxen sodium *(mg/mL)*
F1CGP	1	6	10
F2CGP	1.2	6	10
F3CGP	1.3	6	10
F4CGP	1.4	6	10
F5CGP	1.5	6	10
F6CGP	1.75	6	10
F7CGP	2	6	10

#### Physicochemical Evaluation of Solutions and Gels

Different physicochemical parameters (i.e., clarity, injectability, *in-situ* gelation time, sol–gel fraction, drying ratio, hydration ratio, entrapment efficiency, loading capacity) of prepared solutions and gels were evaluated in accordance with the protocol reported earlier by the authors [26]. Moreover, drug-excipient compatibility, thermal and phase analyses of raw ingredients as well as prepared formulations were conducted by attenuated total reflectance-Fourier transform infrared (ATR-FTIR) spectroscopy, differential scanning calorimetry (DSC and powdered X-ray diffraction (*p*XRD), respectively.

#### *In-Vitro* Drug Release Studies of Chitosan/β-glycerol Phosphate (CGP) Gels

A calibration curve for Naproxen sodium was plotted using PBS (pH 7.4) as the solvent. For this purpose, 50 mg of the drug was accurately weighed and dissolved in a small volume of PBS, and the solution was then diluted to 50 mL in a volumetric flask to obtain a stock solution with a final concentration of 1 mg/ml, designated as first stock solution. Next, 10 mL of this stock solution was transferred to 100 mL volumetric flask and diluted with PBS to achieve a final concentration of 100 μg/mL, designated as the second stock solution. Serial dilutions of this second stock were then prepared to yield concentrations of 10, 20, 40, 60 and 80 μg/mL. The absorbance of each dilution was recorded at 331 nm, which was determined as the maximum wavelength (*λ*_max_) for naproxen sodium by scanning the second stock solution over the full UV range using a UV–Visible (UV–Vis) spectrophotometer. A calibration graph of absorbance *versus* concentration was plotted, from which the regression coefficient (R^2^) and y- intercept were calculated from the linear graph. The limit of detection (LOD), limit of quantification (LOQ) and standard error (SE) were also calculated from the calibration curve.

The drug release investigations across the dialysis membrane were conducted in a modified rotating basket dissolution device with 250 mL PBS pH 7.4 as the dissolving medium and 37 ±  1 °C as the operating temperature. The prepared gel formulations (F5CGP and F6CGP) were sealed in a dialysis tubing (MWCO: 14 kDa, length: 6–8 cm, diameter: 1.43 cm) and placed in the basket, which was positioned on the dissolution device and rotated at 25 rpm. These changes were made to eliminate the peristaltic movements associated with gastrointestinal delivery and confine our formulation to a specific area, i.e. the synovial cavity. To maintain the sink conditions, 3 mL of the dissolution medium was withdrawn and replaced with fresh buffer solution at predefined time intervals of 0.5, 1, 2, 3, 4, 5, 6, 7, 8, 12, 16, 20, 24 h. All experiments were carried out in triplicate, i.e. *n* = 3. The samples were subjected to ultraviolet–visible spectroscopy (UV–Vis) analysis to determine drug concentration at a specific time using a standard curve of pure NAP in PBS pH 7.4.

To describe the gel matrix and understand the kinetics and mechanisms of drug permeation, the *in-vitro* drug permeation data was analysed using various kinetic models (zero order, first order, Higuchi, Korsmeyer-Peppas, and Hixon-Crowell).

#### *In-Vivo* Anti-inflammatory Activity of Naproxen Sodium Loaded, Chitosan/β-glycerol Phosphate (CGP) Gels

The anti-inflammatory activity of NAP-loaded chitosan-GP gels was determined by measuring changes in the diameter of inflamed Sprague–Dawley rats' hind ankle joints after carrageenan injections [40–42]. Briefly, healthy Sprague–Dawley rats of either sex, with average weight between 100–150 g, ± 20 g, were divided into five groups (A: normal, B: positive control, C: placebo or blank formulation, D: test formulation F6CGP and E: negative control) each containing 3 rats. The animals were kept in the same location for 7 days, with a 12-h day-night cycle at 25 ± 2°C. Rats had ad *libitum* access to water and food. The current study was carried out in accordance with the Ethical Committee for the Use of Laboratory Animals guidelines and was approved by the Faculty of Pharmacy, Bahauddin Zakariya University, Multan, Approval no: 09/PEC/2019, which established the rules and regulations for animal research ethics in accordance with the NIH guidelines for the care and use of laboratory animals.

Group A was kept normal throughout the study as no edema was induced. In groups B to E, edema of hind ankle joints was induced by intra-articular injections of carrageenan (1% w/v) daily for 14 consecutive days, administered using a 28-gauge insulin syringe [43]. The difference in the diameter of the ankle joints was recorded to measure the swelling degree as shown in Eq. 1.

1 $$Swelling\;Degree={D}_{2}-{D}_{1}$$

where, *D*_*1*_ is the diameter of the ankle joint before drug or carrageenan administration and *D*_*2*_ is the diameter of the right hind paw ankle joint after drug or carrageenan administration.

Four sets of formulations were created to test the anti-edema activity of gels *in-vivo*. The positive control formulation, a 1% w/v sterile solution of NAP, was administered hypodermically into the swollen region of each rat's right hind ankle joint. Similarly, group C was administered hypodermically with injection of placebo formulation without drug (0.1 mL) at the inflamed region in order to achieve to facilitate gel formation. Each rat in group D was injected with test formulation F6CGP whereas group E was administered with negative control i.e. sterile water for injection only. The diameters of the right hind ankle joints were determined at 3, 6, 9, 12, 24, 48, and 72 h respectively, after drug administration, using a vernier caliper. The reduction in the swelling degree was calculated using Eq. 9.

At different stages of the experiment, digital photographs were captured using a Nikon Coolpix A100 (20 megapixels camera) was used to document the increased swelling and subsequent reduction in the hind ankle joints of all the groups. The photos were taken on the first day of the experiment for normal joints, on the 14th day after carrageenan injection for the most inflamed joints, and finally on the 24th day following formulation injections to document a decrease in inflammation.

Rats were anesthetized by direct isoflorane exposure using Bayne system for a few seconds. Computerized X-ray radiographs of the hind paws of rats were also recorded 3 h after the administration of drug solutions by intra-articular injections for further confirming gel formation under *in-situ* conditions. Radiographs were obtained using digital equipment Fuji film T2 system at 70 kVp and 20 mAs with an exposure time of 0.05 s. The radiographs of all hind paws were recorded medio-laterally (ML) and ventro-dorsally (VD) respectively.

## Results and Discussions

### Physicochemical Evaluation of Solutions and Gels

Visual inspection (Table 2) results indicated that all solutions were very clear to milky white, with no particulate detected in the chitosan-βGP (CGP) solutions. The clarity of the polymeric solution decreased as the polymer concentration in the solution increased.

**Table 2:** Evaluation of polymeric (CGP) solutions

Formulation codes	Chitosan conc MMW (%w/v)	Clarity*	Injectability (for 1 mL soln; sec.) (*n* = 3, SD)
			By assembly
F1CGP	1	+ + +	2.5 (± 0.352)
F2CGP	1.2	+ + +	2.65 (± 0.321)
F3CGP	1.3	+ + +	2.76 (± 0.218)
F4CGP	1.4	+ +	2.83 (± 0.129)
F5CGP	1.5	+ +	2.95 (± 0.536)
F6CGP	1.75	+	3.56 (0.195)
F7CGP	2	+	nil

* + + + = very clear, + + clear, + acceptable milky white clarity

The injectability test showed that all the formulations were injectable and were able to flow through the syringe as the injection time for per mL remained constant for all formulations, except F7CGP which was unable to pass from syringe due to its high viscosity (Table 2).

The results of gelation time, as shown in Table 3, demonstrated that gel formation occurred within 30 min for preparations F5CGP, F6CGP, and F7CGP. In contrast, formulations F1CGP to F4CGP did not form gels within 30 min when placed in a water bath at 37°C. The thermal gelation process started immediately within 5 min. after placing the polymeric and cross-linker solutions at 37°C. All the solutions remained as sol. at room temperature and were converted to gels at 37°C. The gelation time decreased with increasing concentrations of the polymeric solutions. As an organic salt, β-GP exhibited a consistent gelation rate with chitosan solution at neutral pH and 37°C. The phosphate anionic moiety in β-GP likely contributes to gelation onset and is activated at higher temperature to combine with the cationic group of chitosan resulting in ionic crosslinking of chitosan chains. Hence the increasing polymeric concentration resulted in stronger gel structure formation. An advantage that organic salts like glycerol phosphate pose over inorganic salts when used as local *in-situ* crosslinkers is their safety and good pharmacokinetic characteristics and they do not cause any irritation or tissue or cell necrosis [44, 45].

**Table 3:** Evaluation of polymeric (CGP) injectable gels

Sample code	Gelation time (mins) (*n* = 3, SD)	Gel fraction (%) (*n* = 3, SD)	Entrapment efficiency (%) (*n* = 3, SD)	Drug loading capacity (%) (*n* = 3, SD)
F1CGP	–	47.7 (± 0.22)	–	–
F2CGP	–	47.6 (± 0.53)	–	–
F3CGP	–	59.4 (± 0.16)	–	–
F4CGP	–	67.3 (± 0.29)	–	–
F5CGP	23.0 (± 0.54)	91.5 (± 0.79)	50.1 (± 0.22)	8.8 (± 0.04)
F6CGP	17.5 (± 0.89)	92.2 (± 0.81)	70.2 (± 0.52)	8.6 (± 0.03)
F7CGP	12.9 (± 0.67)	94.7 (0.82)	72.2 (± 0.82)	8.3 (± 0.04)

The sol–gel fraction analysis of gels (Table 3) revealed that gel fraction was higher in formulations containing a higher concentration of polymers, with the highest in F5CGP, F6CGP and F7CGP respectively ~ 93 ± 0.519%.

**Fig. 2:**
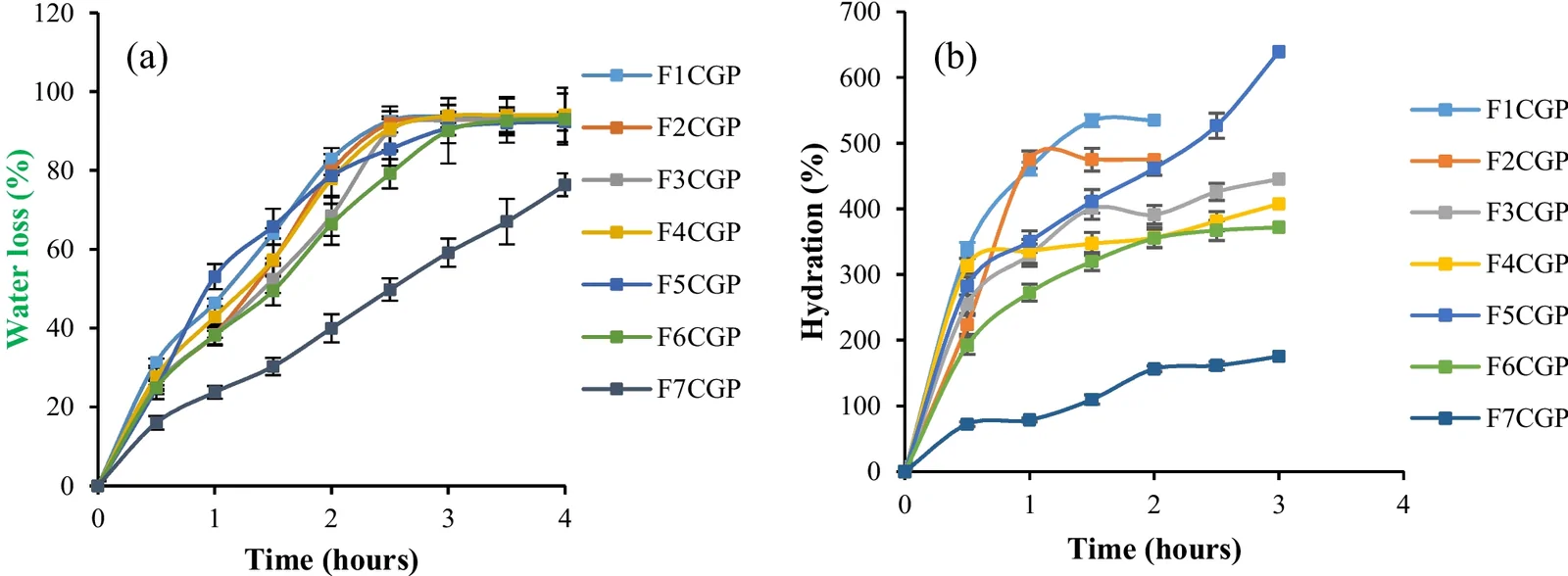
(**a**) Drying behavior of chitosan-βGP gels at 50 °C and pH 7.4. (**b**). Hydration behavior of chitosan-βGP gels at 50 °C and pH 7.4.

The drying behaviours of chitosan-β-GP *in-situ* gels are shown in Fig. 2(a). The gel with a low polymeric concentration (F1CGP through F4CGP) dried at a faster rate in comparison to the counterparts (F5CGP, F6CGP and F7CGP) with higher chitosan concentrations. The water loss from the preparations followed first-order kinetics (R^2^ = 0.9750 ± 0.2). These results indicate that higher polymer densities enhance water binding. Formulation gels F1CGP, F2CGP, F3CGP and F4CGP lost most of their bound water (92.6% ± 1.21, 92.04% ± 1.00, 90.09% ± 5.10, and 90.53 ± 4.61%, respectively) within the first 2.5 h. In contrast, gels F5CGP, F6CGP and F7CGP required 3.5 to 4.5 h to lose a similar amount of water (92.034 ± 3.75%, 92.631 ± 8.30% and 86.12 ± 2.91%, respectively). Higher molecular weight, polymeric concentrations, neutral pH 7.4 and temperature 37 °C contributed in the stronger gel formation. Additionally, the gradual ionization of glycerol phosphate in water contributed to the formation of a highly crosslinked gel matrix and increased the water retention capacity due to induction of linkages between the anionic phosphate and cationic chitosan functional groups and vice versa. It could contribute to modifying the release rate of hydrophilic drugs from chitosan/β-GP gels.

The hydration behaviours of chitosan-βGP xerogels, previously prepared at 37 °C, are shown in Fig. 2(b). The hydration capability was increased by increasing the polymeric concentrations. The rates of liquid-uptake for gels F1CGP and F2CGP (Fig. 2(b)) were higher i.e. 534.2 ± 9.7% and 475 ± 13.2% respectively as compared to the rest of the gels, when soaked in phosphate buffer pH 7.4 for 1.5 h at 50°C. Afterwards, the gels collapsed owing to weaker integrity. Whereas, for gels F3CGP and F4CGP (Fig. 2(b)), percentage of hydration was 400.1 ± 17.2% and 347 18.21%, respectively within 1.5 h. Afterwards, a gradual increase was observed in hydration till 3 h. However, this increase was not substantial and stopped at 445 ± 12.9% and 407.7 ± 14.8% respectively because of low optimal polymeric concentrations and weaker nature of gels formed. The formulations F1CGP to F4CGP eroded after 3 h and hence, were excluded from further studies. For the gels, F5CGP, F6CGP and F7CGP, water reuptake capacity was 411.5% ± 16.3%, 319.8% ± 12.9% and 109.7 ± 2.8% respectively in 1.5 h. The hydration capacity maximized i.e., 639 ± 19.2%, 372 ± 15.2% and 175 ± 6.2%, respectively after 3 h. The water uptake capacity of F7CGP was the lowest likely due to higher polymeric concentration and increased viscosity. Hence, it was also not suitable for further studies. Gel formulations F5CGP and F6CGP depicted a gradual increase, and remained stable during the hydration as their graph curves were less steep than their counterparts. It was evident that the maximum % hydration of F6CGP and F7CGP (Fig. 2(b)) was less than F1CGP, F2CGP, F3CGP, F4CGP and F5CGP after 1.5 h. This difference was attributed to the relatively more hydrophobic 3D crosslinked gel structure of F5CGP, F6CGP and F7CGP owing to their higher polymeric content.

The EE and LC of formulations F5CGP, F6CGP and F7CGP were found to be 50.1 ± 0.22%; 8.8 ± 0.04% and 70.2 ± 0.52%; 8.6 ± 0.03% and 72.2 ± 0.82%; 8.3 ± 0.04%, respectively (Table 3). Gel formulations containing higher concentrations of chitosan exhibited greater EE and LC than those with lower concentrations ones. This increase may be attributed to the higher molar mass and polymer density, which provide greater stability and structural integrity to the cross-linked gel network.

**Fig. 3:**
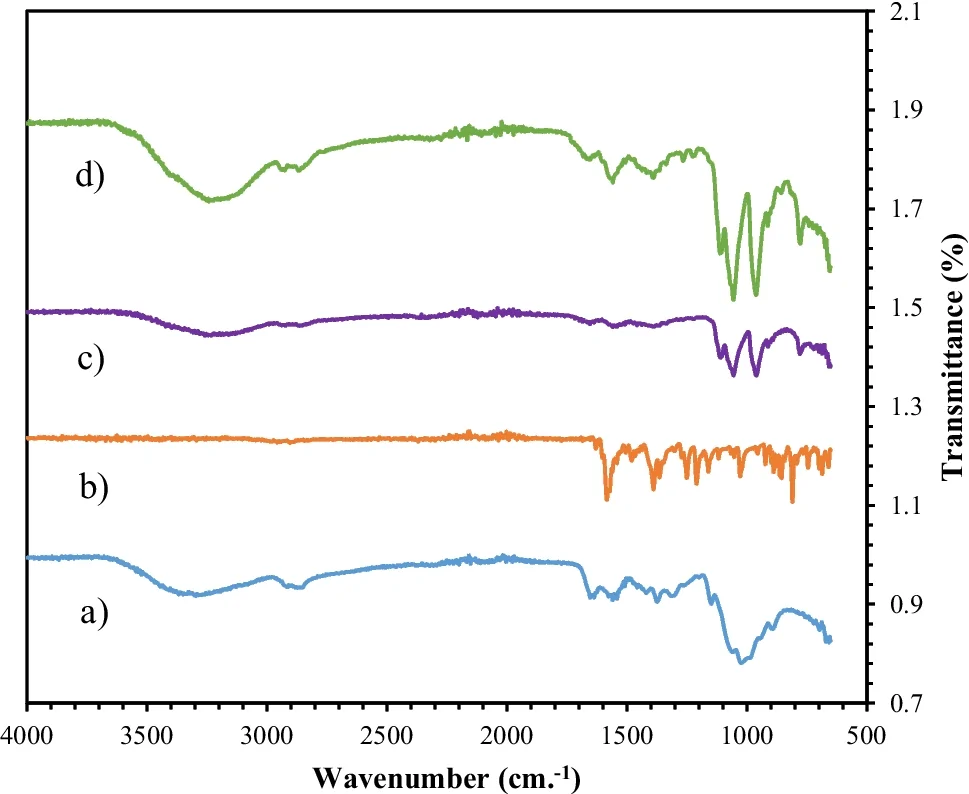
Fourier transform infra-red (FTIR) analysis of; (**a**) Pure chitosan powder MMW (CHIT), (**b**) Naproxen sodium (NAP), (**c**) Empty gel formulation F6CGP without drug, (**d**) NAP sodium loaded formulation F6CGP.

The FTIR spectrum of chitosan (Fig. 3a) indicate a sharp absorption peak between 2791–2922 cm^−1^ corresponding to -COOH stretching. A broad peak at region 3000–3500 cm^−1^ region was associated with strong –OH and medium –NH stretching. Peaks at 1632 and 1545 cm^−1^ indicated symmetric and asymmetric -C = O- and weak -C = C- aromatic stretching vibrations. A shoulder peak at 1151 cm.^−1^ represented -C–O–C stretching, while peaks at 1019 and 1053 cm.^−1^ correspond to -C–OH stretching vibration in chitosan structure. A distinct peak at 652 cm^−1^ was attributed to out of the plane –NH bending [46]. FTIR spectrum for pure naproxen sodium (NAP) Fig. 3(b) illustrates an aliphatic -CH stretch at 2900 cm^−1^, a strong aromatic –C = C stretch at 1582 cm.^−1^, and peaks at 1388 cm^−1^ depicted a strong –C–O–C stretching and –CH bending. FTIR spectrum of dried chitosan/β-GP, empty gel formulation F6CGP (without drug) has been shown in Fig. 3(c) exhibited an absorption band at 3500–2937 cm^−1^ indicating an existence of strong –OH stretch. A characteristic peak around 1556 cm^−1^ due to C = O aromatic stretch and characteristic peaks at 1054 and 1106 cm^−1^ respectively due to strong –C–OH stretches. The spectrum of NAP-loaded dry gel (F6CGP) crosslinked with β glycerol phosphate at 37 °C (Fig. 3d) indicate a broader band at 3500–3100 cm.^−1^ (–OH stretching) and a peak at 2900 cm^−1^ (–COOH stretching). Distinct peaks at 1054 and 1105 cm^−1^ indicated –CO and –C–OH linkages from Naproxen sodium and chitosan, a peak at 1389 cm^−1^ corresponded to medium –OH bending of phenolic group of NAP.

**Fig. 4:**
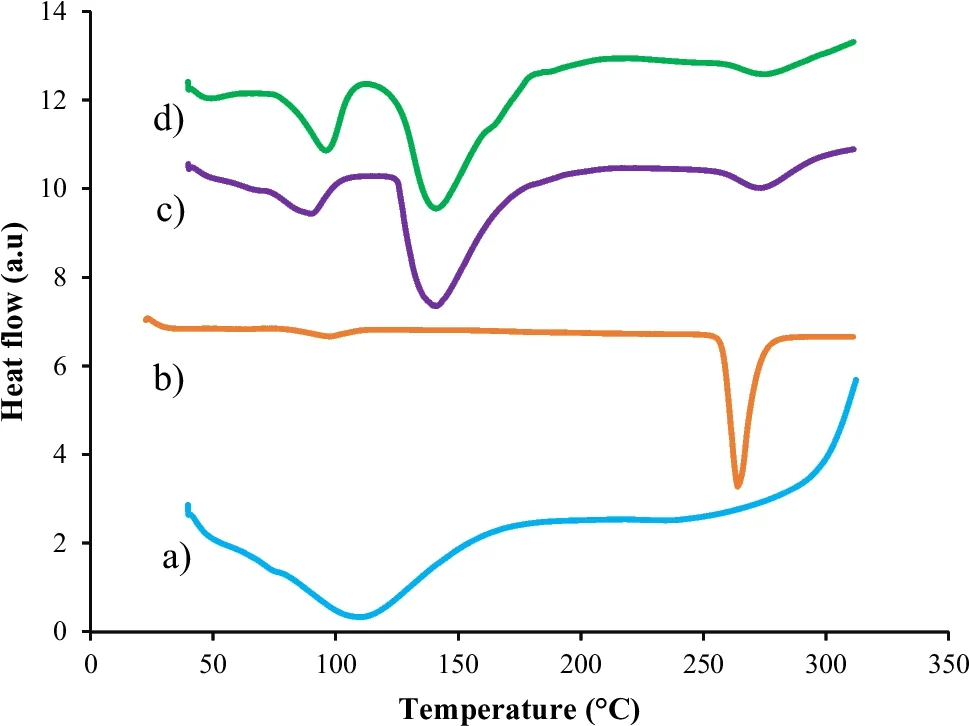
DSC thermograms for (**a**) chitosan MMW (CHIT), (**b**) pure naproxen sodium (NAP (**c**) empty dried formulation F6CGP, (**d**) NAP loaded gel formulation F6CGP.

The DSC thermogram of chitosan (Fig. 4a) showed a broad endothermic peak between 75 °C and 150 °C due to loss of water coupled with glass transition. An exothermic peak initiating after 300 °C indicated the decomposition of carbohydrate polymer chitosan [47]. For pure naproxen sodium (NAP) (Fig. 4(b)), a slight endothermic dip observed at 90–105°C was attributed to the loss of water via evaporation. Another sharp endothermic peak was recorded at around 263 °C (enthalpy of 118.9 J/g), depicting the melting of pure crystalline drug (melting point of NAP; 260 °C). The DSC thermogram of chitosan/β-GP dried empty gels (F6CGP) (Fig. 4(c)) showed a broad endotherm at 78 and 102 °C owing to the loss of unbound water. Similarly, a broad endothermic peak between 125 °C to 170 °C was possibly due to the dehydration of phosphate pentahydrate coupled with glass transition of gel. An endotherm after 260 °C suggested decomposition of gel. Thermogram of NAP loaded gel F6CGP (Fig. 4(d)) depicted endothermic peaks at 80–105°C and 125–170°C due to the removal of water and dehydration of unreacted β-glycerol phosphate pentahydrate coupled with glass transition of gel, respectively. A broad endotherm after 260 °C suggested decomposition of chitosan-βGP gel (Fig. 4(d)). Distinctive melting peak of drug was absent from the DSC scan of drug loaded gel formulation indicating its reduced crystallinity.

**Fig. 5:**
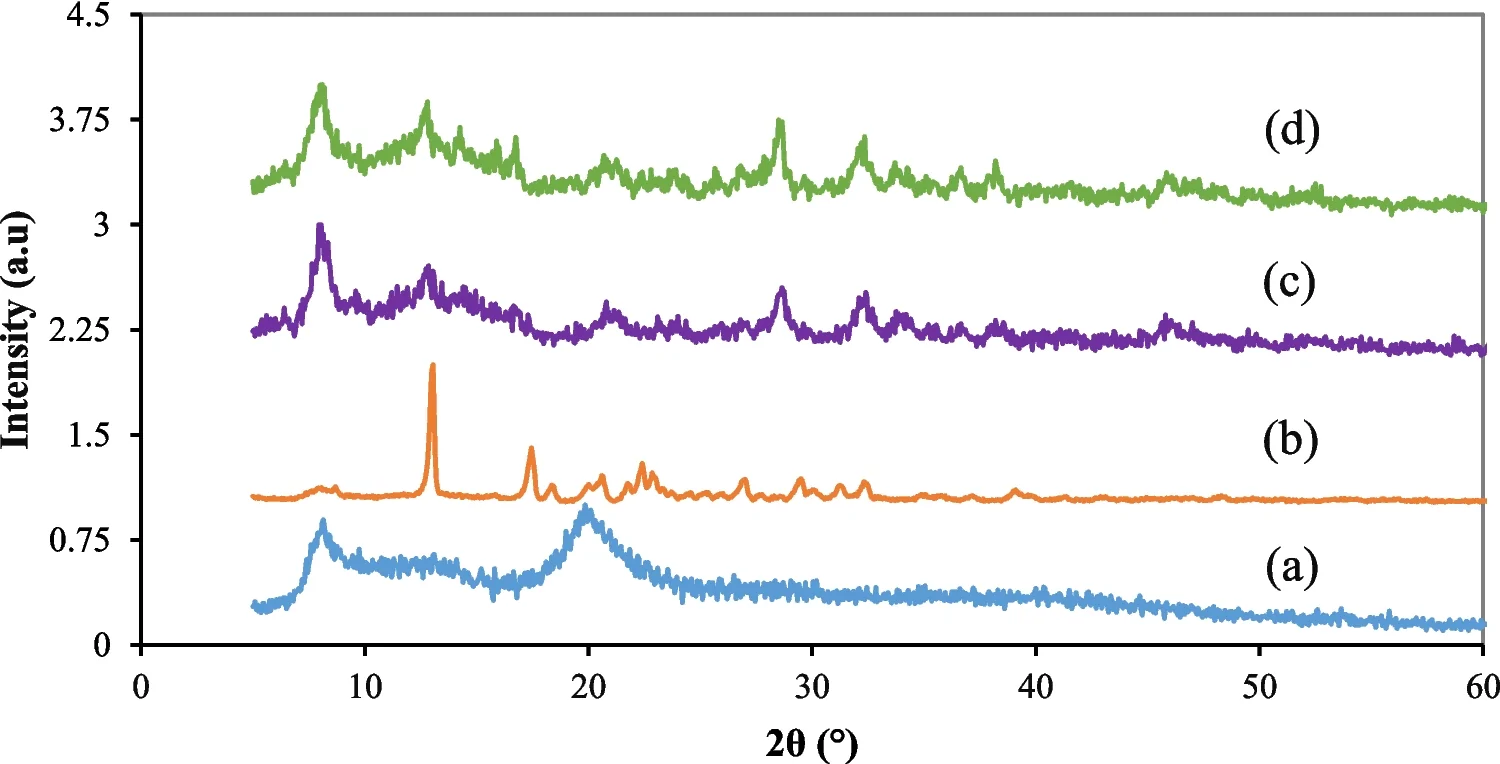
*p*XRD diffractograms of (**a**) chitosan (MMW) (CHIT), (**b**) pure naproxen sodium (NAP), (**c**) dried empty formulation F6CGP and (**d**) NAP loaded dried formulation F6CGP.

The *p*XRD results of chitosan, NAP, F6CGP (gel without drug) and F6CGP (gel with drug) are shown in Fig. 5. The X-ray diffractogram of pure chitosan powder (Fig. 5a) shows broad peaks at 2θ = 9.01° and 21°, indicating its semi-crystalline and polymorphic nature of chitosan. Similarly, the X-ray diffractogram of NAP (Fig. 5(b)) exhibit characteristic crystalline peaks at 2θ = 13.05°, 17.45°, 20.6° and 23.1°. The X-rays diffraction pattern of empty gel formulation without drug F6CGP (Fig. 5(c)) depicts characteristic diffraction peaks at 2θ = 8.15°, 13.1°, 21.35°, 28.65 and 32.25° respectively, confirming the semi-crystalline and polymorphic nature of the gel. In contrast, the NAP-loaded dried F6CGP gel (Fig. 5d) shows peaks at 2θ = 8.15°, 12.75°, 28.6° and 32.2°, with a broad Diffraction peak recorded at 12.75° suggests the semi-crystalline nature of chitosan-based matrix. The Characteristic sharp peaks relating to crystalline nature of NAP were not observed in F6CGP diffractogram, suggesting a phase transition of NAP in the course of formulation of NAP during gel formulation and drying. These findings are consistent with the DSC results.

### *In-Vitro *Drug Release Studies of Chitosan/β-glycerol Phosphate (CGP) Gels

A linear calibration curve with a slope and R^2^ of 0.0058 and 0.9996, respectively was successfully established. The LOD (detection limit) and LOQ (quantification limit) were recorded to be 2.064 μg/mL and 6.254 μg/mL, respectively.

**Fig. 6:**
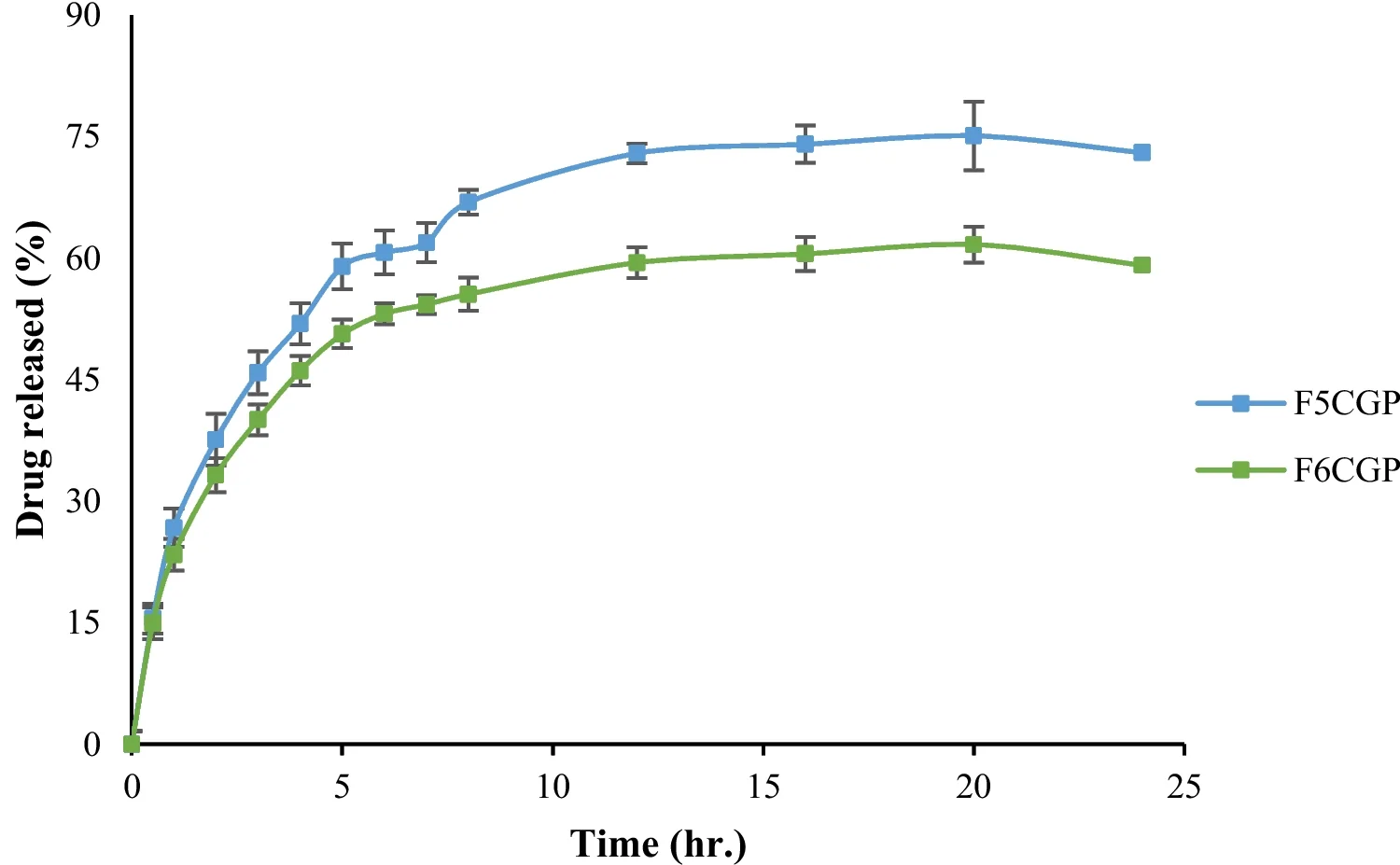
Drug release profiles of formulations (F5CGP and F6CGP) through the dialyzing membrane at 37 °C in PBS (pH 7.4).

Figure 6 depicts the drug release profiles of various formulations. Within the first 5 h, F5CGP released 58.965 ± 2.52% of the drug into the dissolution medium, after which the release reached a plateau (~ 75% within 20 h). The incomplete drug release (75% within 20 h) was possibly due to the entrapment of naproxen sodium molecules within the crosslinked gel network. The polymeric matrix likely restricted drug diffusion leading to a slower release. The remaining naproxen sodium might continue to release gradually over an extended time period as the gel network undergoes structural changes leading to sustained release behavior. In comparison, F6CGP released relatively lower amount of drug i.e., 50.63 ± 1.837% over the same period due to higher gel fraction (Table 3). Both formulations exhibited non-linear release profiles during the initial phase, which may be attributed to the abundance of drug molecules on the gel surface and within the outer gel layers, their weak entanglements with the hydrogel matrix, and their close proximity to the dissolution medium. Subsequently, the formulations exhibited sustained release due to the formation of stronger drug-polymer entanglements within the gel network, resulting in a reservoir-like matrix structure. The overall release rate of F6CGP was lower than that of F5CGP, which can be described to its lower porosity and more viscous nature of F6CGP. The Korsmeyer-Peppas kinetic model provided the best fit for both F5CGP and F6CGP with *n* = 0.291 and 0.261 and R^2^ = 0.949 and 0.9330, respectively. The relatively slower and more sustained release of F6CGP is likely due to the formation of a more stable gel at higher polymeric concentrations. As the drug molecules diffused through the gel matrix, the value of *n* ≤ 0.45 confirmed a Fickian diffusion-controlled release mechanism [10]. Based on its superior sustained-release profile, F6CGP, was selected for further evaluation.

### *In-Vivo* Anti-Inflammatory Activity of Naproxen Sodium Loaded, Chitosan/β-glycerol Phosphate (CGP) Gels

**Fig. 7:**
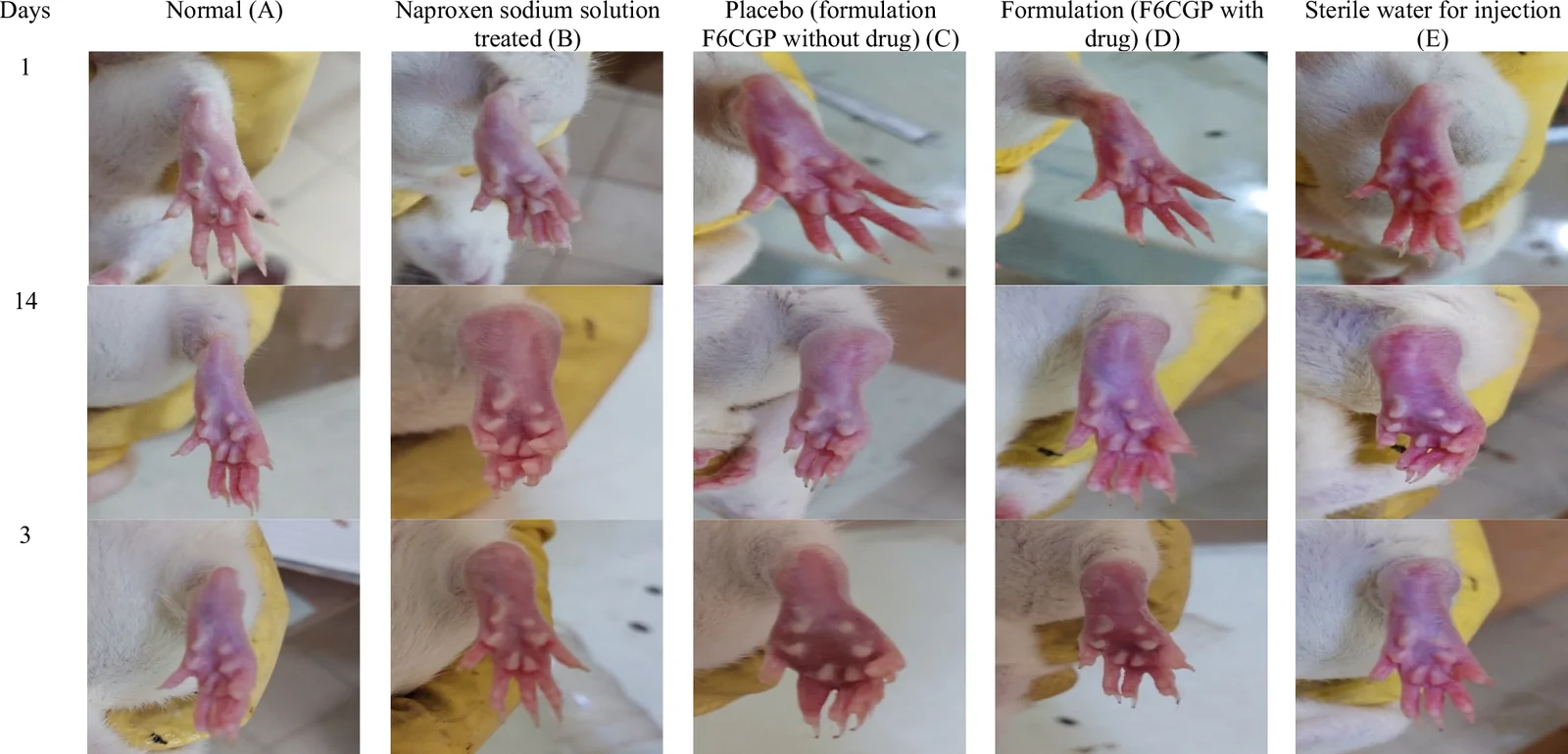
Images of rats right hind paws before carrageenan injection (at day 1), after carrageenan-induced inflammation (at day 14) and after formulation F6CGP injections (at day 3).

**Fig. 8:**
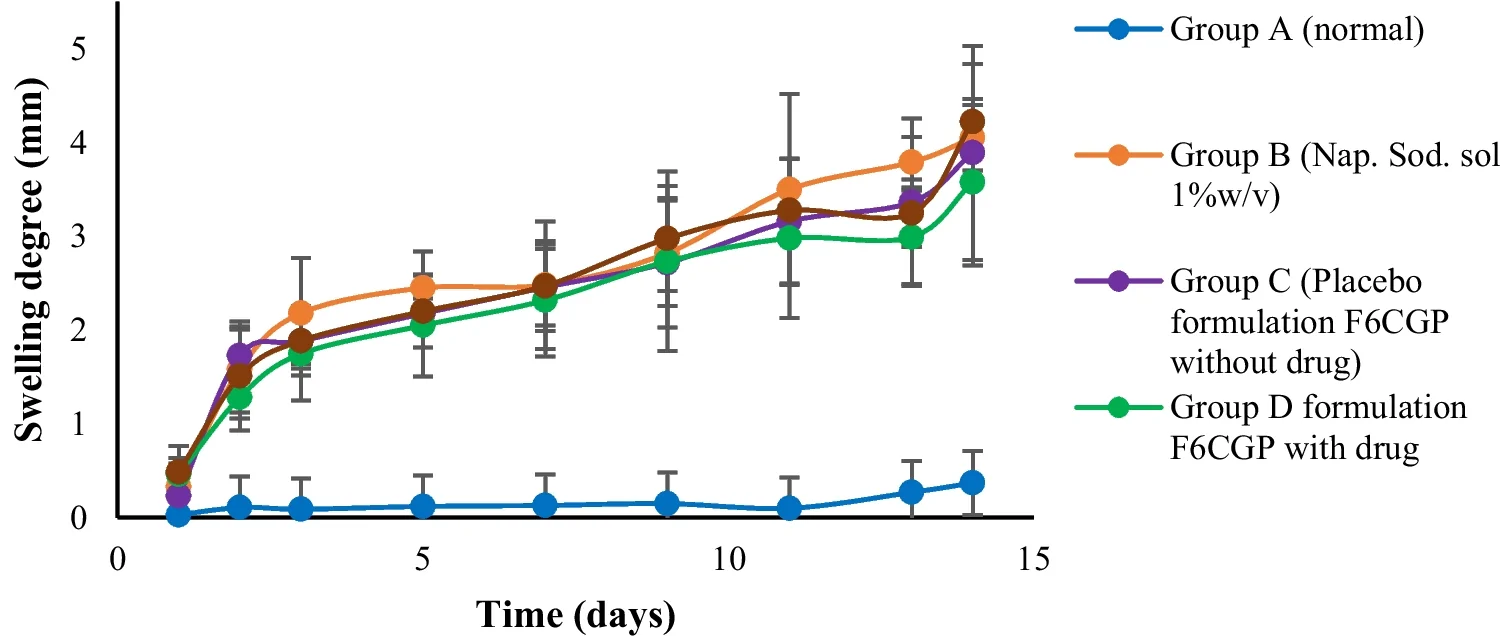
Swelling degree increase in rats groups A, B, C, D and E after treatment with carrageenan.

Swelling of rat hind ankle joints following administration of carrageenan for 14 days is described in Fig. 7 and Fig. 8. An increment in diameter of right hind ankle joint, 4.05 mm ± 0.35 mm, 3.89 mm ± 1.14 mm, 3.58 mm ± 0.89 mm and 4.22 mm ± 0.61 mm was recorded in groups B, C, D and E, respectively. This confirms the establishment of inflammatory model. The diameter of the normal group (A) remained constant at 6.47mm throughout the study.

**Fig. 9:**
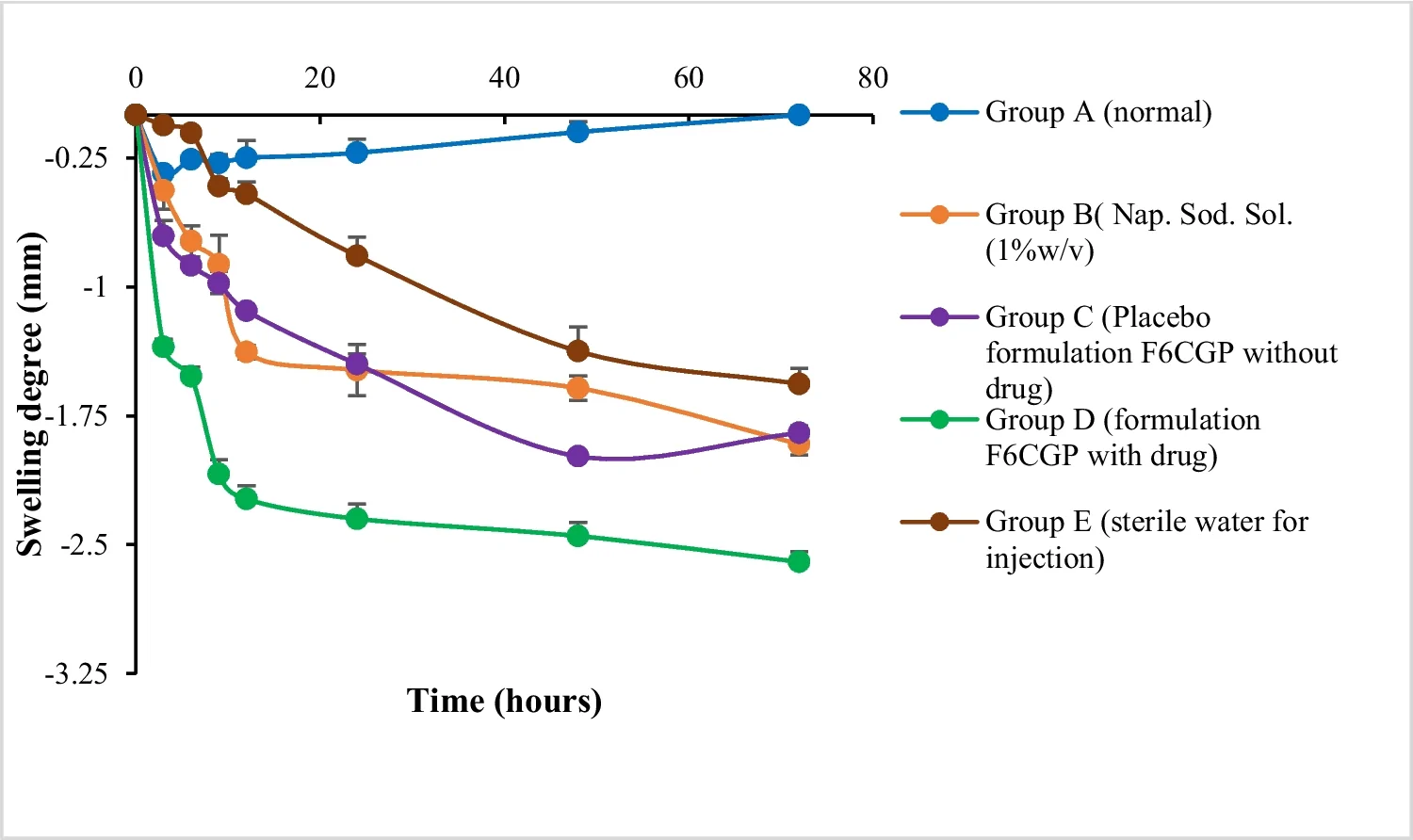
Swelling degree reduction rats groups A, B, C, D and E after treatment with formulations.

**Fig. 10:**
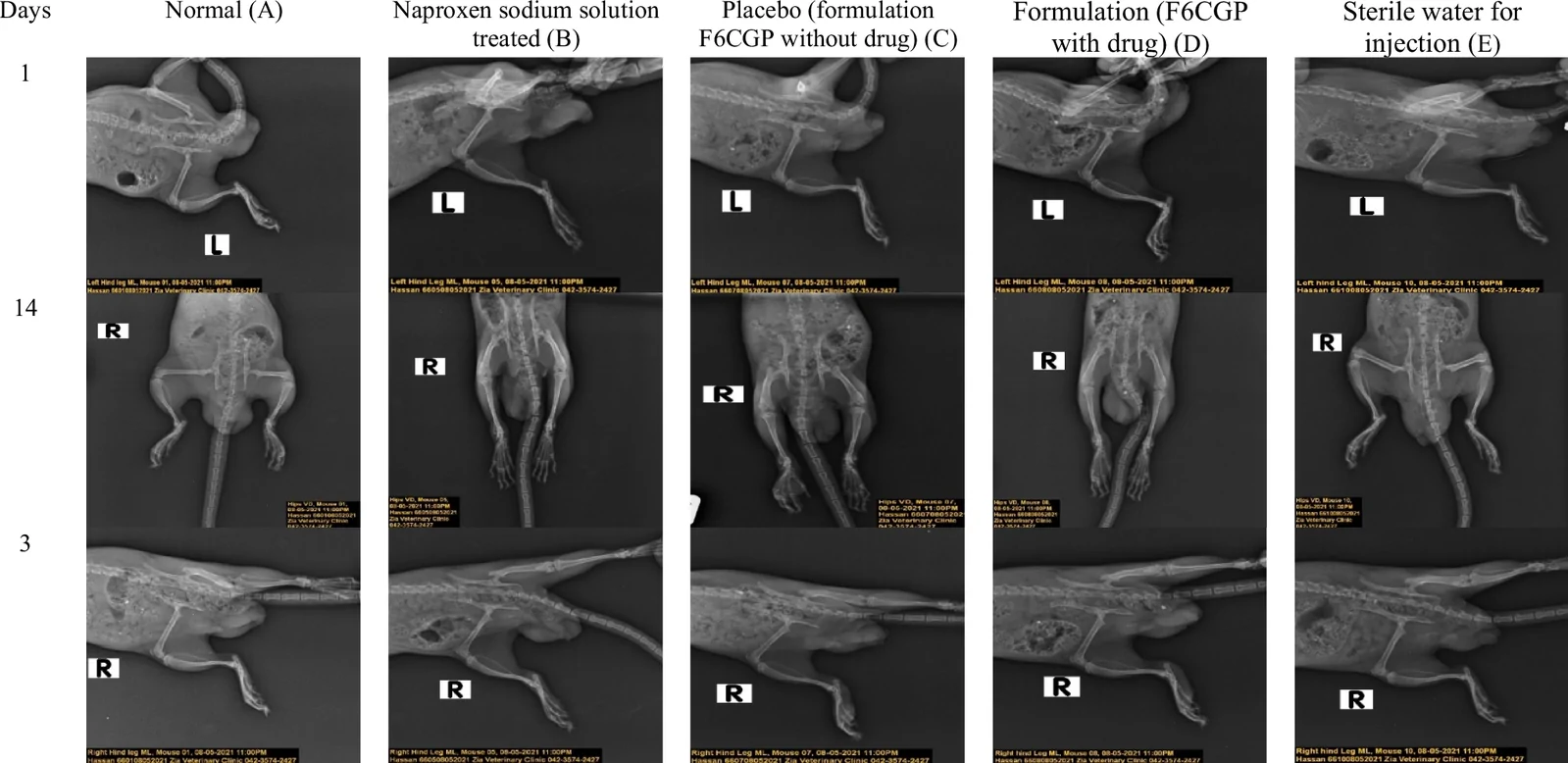
X-ray radiographs of rats hind paws of normal (no inflammation) and different treatment groups before carrageenan injection (at day 1), after carrageenan-induced inflammation (at day 14) and after formulation F6CGP injections (at day 3). Images at day 1 are medio-lateral (ML) views of left hind paws. Images at day 14 are ventro-dorsal (VD) views of both hind limbs. Images at day 3 are medio-lateral (ML) views of right hind paws.

During the next stage, changes in right hind ankle joints were recorded following the administration of formulations (Fig. 7 and Fig. 9). At 24 h, a reduction of 1.485 ± 0.195 mm, 1.45 ± 2.27 mm, 2.35 ± 0.87 mm and 0.82 ± 0.76 mm was recorded in groups B, C, D and E respectively. Over the course of an additional 48 h, a consistent reduction in the swelling of the hind paw ankle joint (2.6 ± 0.73 mm) was observed in group D. This demonstrates the gel formulation's ability to provide sustained drug release. The rats in groups E and B, in contrast, did not show any further reduction in swelling over the next 48 h due to the disposition of drug from the site of action. In summary, after 72 h, the reduction in swelling recorded in various groups follows the order; groups D > C > B > E. The blank chitosan/β-GP *in-situ* gelling formulation (F6CGP, group C) led to a reduction in swelling likely due to the immunological properties of polymer. Chitosan is reported to inhibit pro-inflammatory cytokines [48], promote tissue granulation through fibroblast recruitment [49] and produce type III collagen [50]. In addition, the gel matrix possibly provided a protective local environment which facilitated reduction in swelling. Nevertheless, the drug loaded formulation exhibited relatively higher reduction in swelling due to the pharmacological inhibition of cyclooxygenase-mediated prostaglandin synthesis by naproxen sodium. Furthermore, the evidence of *in-situ* gel formation was further supported by X-ray images of the rat hind paws (Fig. 10), which showed a distinct opaque region around the ankle joint area in rats receiving the F6CGP formulation, with or without drug.

## Discussion

In the present study, a naproxen sodium loaded β-GP crosslinked chitosan-based *in-situ* gelling drug delivery system was developed and characterized. Here, the prepared formulation is compared with previously reported naproxen sodium loaded calcium gluconate crosslinked sodium alginate *in-situ* gel [26]. These two *in-situ* gelling systems differed in their base polymers and crosslinking mechanisms. Chitosan is a cationic polymer soluble in an acidic environment. β-GP facilitates thermosensitive gelation of chitosan at ~  37 °C by reducing its solubility/protonation of its amino groups and forming physical crosslinks, hydrogen bonds as well as electrostatic interactions with the polymer [31, 51, 52]. Sodium alginate, an anionic aqueous soluble polysaccharide rapidly undergoes ionic gelation and forms a three-dimensional (3D) network following interaction with a divalent cation i.e., calcium [53]. These differences influenced key features of prepared gels e.g., gelation time, gel fraction, injectability, water reuptake ability, drug release etc.

Gelation time is a critical parameter particularly for injectable *in-situ* gels systems because it determines the time required by the formulation for transforming from sol to gel state at the site of administration. Chitosan-based gel showed a ~ 2.7X longer gelation time as compared to alginate counterpart. Prompt interaction between calcium ions and alginate chains leads to rapid gelation. In contrast, chitosan-based system relies on temperature induced sol to gel transition and physical/ionic crosslinking which are relatively slower in establishing a network, thus, leading to extended gelation times. This would permit more uniform distribution of precursor solution at the site of administration, in turn, homogenous gel matrix. Chitosan-based gel displayed a slightly higher gel fraction (~ 92%) as compared to alginate (~ 89%) suggesting a relatively more homogenous and stable network. Furthermore, chitosan-based formulation exhibited a higher injectability (3.56) than alginate (3.25) possibly due to lower viscosity of pre-gelled solution suggesting that the prepared formulation would be relatively easier to inject.

Chitosan gel exhibited a ~ 5.36X lesser water reuptake capacity following rehydration of xerogel. Swelling ability is influenced by the polymer hydrophilicity, crosslinking density and mesh size of gel network. Hydrophilicity and ionic crosslinking facilitated water absorption by the alginate gel [54]. Chitosan gel absorbed relatively less water due to its degree of deacetylation and compact network. This would preserve gel architecture and allow better mechanical stability of gel *in-vivo*. Chitosan gel showed ~ 2.3fold higher drug release as compared to alginate counterpart. Possibly, physically associated network of chitosan permitted rapid drug release owing to weaker physical entrapment. Gelation of alginate is usually described by the egg-box model. According to this model, calcium forms ionic crosslinks with the guluronic acid blocks of alginate chain to form a 3D structure which resembles eggs arranged in an egg box [55]. This compact egg-box structure possibly reduced the matrix pore size, thus, restricting the diffusion of encapsulated naproxen sodium. A higher swelling ability of alginate might create a diffusional barrier resulting in relatively slower drug release. This rapid drug release and inherent anti-inflammatory properties of chitosan led to a 10% more reduction in rat paw swelling following treatment with chitosan-based gel as compared to alginate gel [56, 57].

These results indicate relatively better performance and therapeutic outcomes by chitosan and β-GP-based *in-situ* gelling system. Moreover, chitosan is degraded mainly by lysozymes present in the body, thus, reducing the risk of long-term accumulation [58]. Conversely, alginate is not metabolized in the mammalian tissues and depends on ion exchange or dissolution for clearance. This process is slower and may leave residual material within the body [59].

Overall, chitosan-based system exhibited a therapeutically favourable profile as compared to sodium alginate counterpart. A longer gelation time would allow the gel to develop in a resilient manner in the cavity fluid [26, 60]. A relatively higher gel fraction remits an improved structural integrity and better injectability supports ease of administration [26, 61, 62]. A relatively rapid drug release and anti-inflammatory properties of chitosan resulted in an improved therapeutic efficacy as evidenced by higher reduction in rat paw edema. Further, lysozyme-mediated degradation of chitosan provides an advantage over alginate in terms of predictable metabolism and excretion. Collectively, these findings establish β-GP crosslinked chitosan-based gel-forming injectable formulation as a superior contender for arthritis treatment.

## Conclusion

Based on the findings, an injectable gel formulation of chitosan/β-GP containing NAP was successfully developed under physiologically simulated conditions using ionic and thermo-gelation at pH 7.4 and 37°C. Gels prepared with higher concentrations of chitosan improved physicochemical properties, including enhanced drying/hydration behaviour and sustained *in-vitro* release profiles, compared to those with lower chitosan content. Among all formulations, F6CGP showed the most favourable characteristics, supporting prolonged swelling and sustained drug release. FTIR analysis revealed H-bonding interactions among chitosan, β-glycerol phosphate, and NAP. DSC and *p*XRD analyses verified the polymorphic and semicrystalline nature of the drug within the polymeric matrix. *In-vivo* anti-inflammatory studies demonstrated a sustained reduction in oedema for up to 72 h when compared with the control group. Overall, the developed *in-situ* injectable gel formulation shows promise for delivering NAP to the articular cavity in a controlled manner, offering potential benefits for arthritis treatment.

## Declaration

### Clinical Trial Number

Not Applicable (no clinical trials).
